# Waking Action of Ursodeoxycholic Acid (UDCA) Involves Histamine and GABA_A_ Receptor Block

**DOI:** 10.1371/journal.pone.0042512

**Published:** 2012-08-06

**Authors:** Yevgenij Yanovsky, Stephan R. Schubring, Quiaoling Yao, Yan Zhao, Sha Li, Andrea May, Helmut L. Haas, Jian-Sheng Lin, Olga A. Sergeeva

**Affiliations:** 1 Department of Neurophysiology, Medical Faculty, Heinrich-Heine-University, Dusseldorf, Germany; 2 Integrative Physiology of Brain Arousal Systems, Lyon Neuroscience Research Center, INSERM U1028-CNRS UMR 5292, Faculty of Medicine, Claude Bernard University, Lyon, France; Indiana University School of Medicine, United States of America

## Abstract

Since ancient times ursodeoxycholic acid (UDCA), a constituent of bile, is used against gallstone formation and cholestasis. A neuroprotective action of UDCA was demonstrated recently in models of Alzheimer's disease and retinal degeneration. The mechanisms of UDCA action in the nervous system are poorly understood. We show now that UDCA promotes wakefulness during the active period of the day, lacking this activity in histamine-deficient mice. In cultured hypothalamic neurons UDCA did not affect firing rate but synchronized the firing, an effect abolished by the GABA_A_R antagonist gabazine. In histaminergic neurons recorded in slices UDCA reduced amplitude and duration of spontaneous and evoked IPSCs. In acutely isolated histaminergic neurons UDCA inhibited GABA-evoked currents and sIPSCs starting at 10 µM (IC_50_ = 70 µM) and did not affect NMDA- and AMPA-receptor mediated currents at 100 µM. Recombinant GABA_A_ receptors composed of α1, β1–3 and γ2L subunits expressed in HEK293 cells displayed a sensitivity to UDCA similar to that of native GABA_A_ receptors. The mutation α1V256S, known to reduce the inhibitory action of pregnenolone sulphate, reduced the potency of UDCA. The mutation α1Q241L, which abolishes GABA_A_R potentiation by several neurosteroids, had no effect on GABA_A_R inhibition by UDCA. In conclusion, UDCA enhances alertness through disinhibition, at least partially of the histaminergic system via GABA_A_ receptors.

## Introduction

Ursodeoxycholic acid (UDCA) and its taurine conjugate tauroursodeoxycholate (TUDC), major constituents of black bear bile, are used for over 3000 years to treat not only liver disorders (such as cholesterol gallstones, cholestasis, sclerotic cholangitis, primary biliary cirrhosis) [1] but also visual system disorders [2], [3]. More recently UDCA was found beneficial in the prevention of neuronal degeneration and apoptosis [4]–[6]. UDCA represents a minor fraction of the human bile acid pool (2–5%), but after several month of UDCA therapy (up to 35 mg/kg) it substitutes for the so called “bad” pro-apoptotic bile acids, such as chenodeoxycholate and deoxycholate and comprises 58–69% of the total bile acid pool exerting a choleretic action and improving liver function [7], [8]. Effects of UDCA on the sleep-wake pattern were not yet investigated, although by combating pruritus UDCA improves the quality of life and of sleep [9]. UDCA and other Bile Steroids (BS stands for both: bile salts and bile acids), beyond digestive function, are signaling molecules with broad paracrine and endocrine functions [10], [11]. We have recently shown that common BS which are synthesized in the brain, such as cholate and chenodeoxycholate, antagonize GABA_A_ and NMDA receptors [12] and can by this way influence hypothalamic control of energy homeostasis.

Histamine, produced in a hypothalamus by the small group of neurons located in the tuberomamillary nucleus (TMN), supports cortical arousal [13] and is involved in the control of feeding behavior and energy expenditure [14]. As a “waking transmitter” histamine counteracts the action of anaesthetics and hypnotics [15]. Firing of histaminergic neurons is under the control of GABAergic input from the ventrolateral preoptic area (VLPO) and other excitatory and inhibitory inputs [14], [16]. A GABA_A_R agonist injected in the TMN region induces sleep [17], whereas a GABA_A_ receptor antagonist not only increases waking but can terminate anesthesia [15], [18].

The present study investigates the action of UDCA on sleep-wake stages of the mouse and compares effects between wild type and histamine deficient mice. A direct antagonistic action of UDCA on GABA_A_R of TMN neurons recorded in slices and after acute isolation suggests that disinhibition of histaminergic neurons supports the wake-promoting action of UDCA. Substances like UDCA may be useful for the treatment of diseases accompanied by an increased GABAergic tone, fatigue and day-time sleepiness such as hepatic encephalopathy [19].

## Results

### UDCA increases waking in wild type but not in histamine-deficient mice

**Figure 1:**
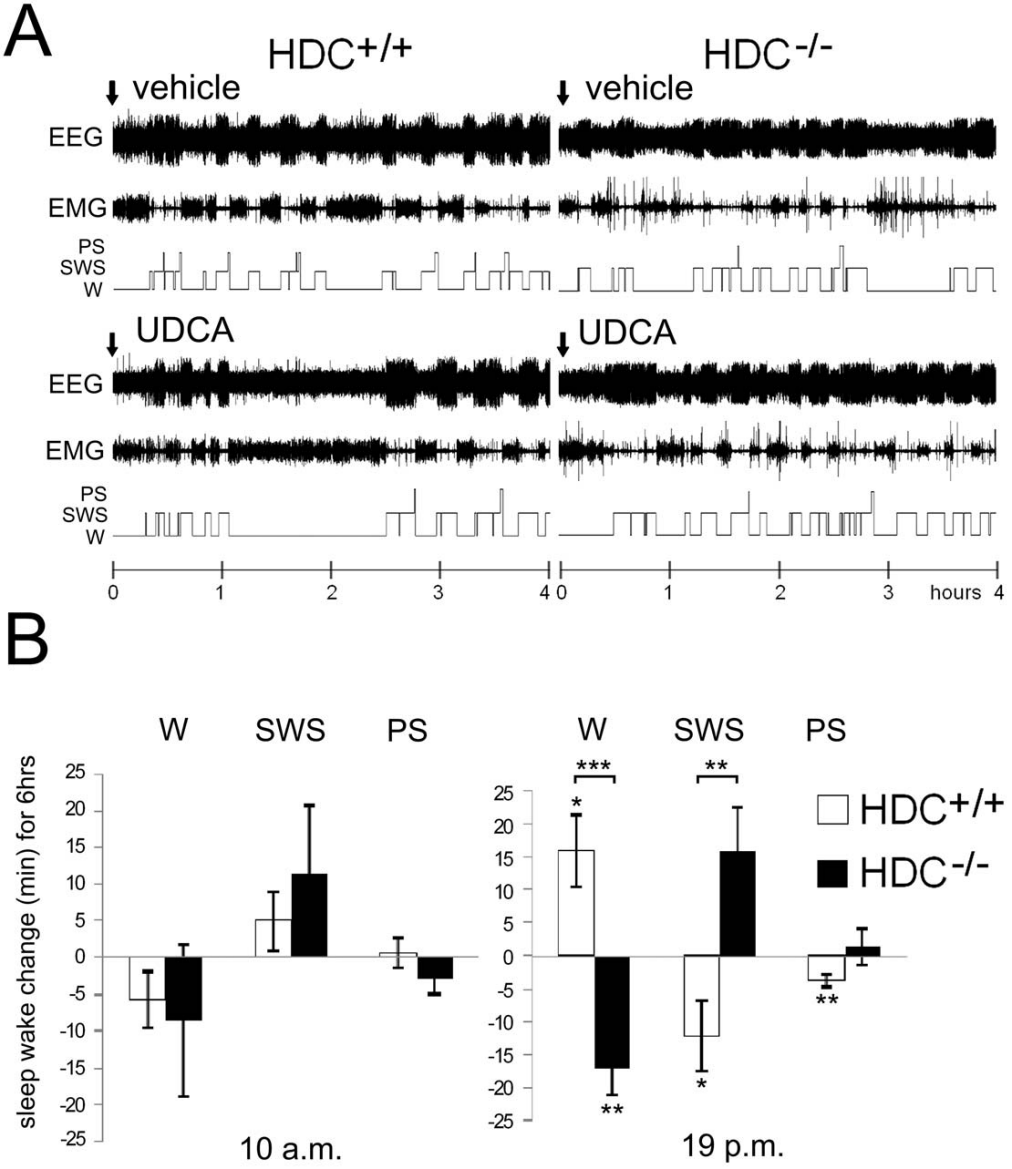
UDCA given orally increases wakefulness. A. Polygraphic recording (EEG and EMG) and corresponding hypnograms showing the effect of UDCA given at 7 p.m. B. Changes in sleep-wake duration after UDCA application relative to control (vehicle application) in wild type (HDC +/+) and histidine decarboxylase deficient (HDC−/−) mice. In both mouse genotypes UDCA given during the sleepy period of the day (10 a.m.) does not affect sleep-wake states, but does so during the active period of the day. Given at 7 p.m. UDCA increases waking (W) and decreases slow wave sleep (SWS) and paradoxical sleep (PS) in the HDC+/+ mice. In HDC−/− mice UDCA decreases waking. The significant difference between time spent in a given vigilance state in control and under UDCA is indicated with stars near the corresponding bars. Significant difference between genotypes is indicated with stars on top of brackets. * p<0.05; ** p<0.01; *** p<0.005.

Given during the active period of the day (7 p.m.) in HDC+/+ mice (n = 11) UDCA significantly increased waking (starting from the third hour after application, p<0.05) and decreased the amount of slow wave sleep (SWS, p<0.05) and paradoxical sleep (PS, p<0.01). In contrast, in histamine-deficient mice waking was significantly decreased by UDCA (p<0.01), whereas total sleep was increased (p<0.01). Slow wave sleep (SWS) and paradoxical sleep (PS) tended to be increased by UDCA in HDC−/− mice in comparison with vehicle control. The change in SWS under UDCA differed significantly between HDC−/− and HDC+/+ mice (Fig. 1B, p<0.01). At 32 mg/kg (maximal clinical dose) UDCA did not affect sleep wake states when given orally during the “quiet period” of the day (10 a.m.) either in wild type (HDC+/+, n = 11)) or histamine-deficient mice (HDC−/−, n = 7) (Fig. 1).

The significant difference between UDCA action in wild type and histamine-deficient mice raised the possibility that this bile steroid (BS) influences the activity of histaminergic neurons.

### UDCA does not increase firing rate of hypothalamic neurons

**Figure 2:**
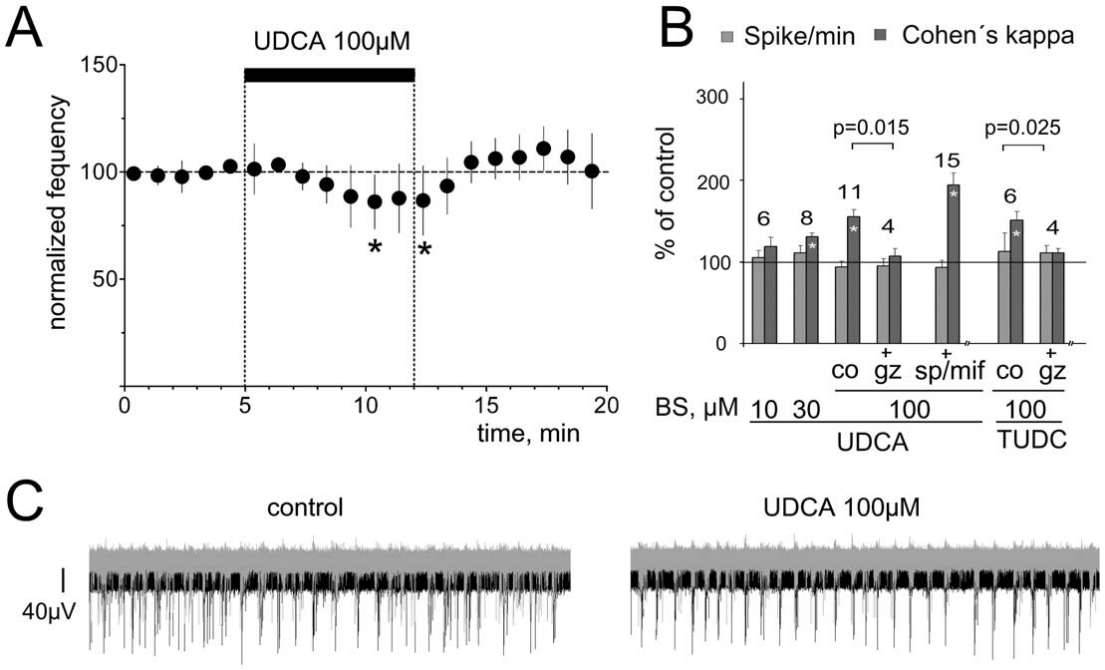
UDCA synchronizes network activity like a GABA_A_ receptor antagonist. **A.** Firing rate of TMN neurons (n = 10) recorded in mouse hypothalamic slices is not significantly affected by UDCA during the first 5 min of UDCA perfusion. Each filled circle represents the average firing during 5 min. Significant difference from baseline is indicated with stars (* p<0.05, Wilcoxon test). **B.** Summary of MEA experiments illustrates the change in spikes/min (all spikes over all active electrodes) and Cohen's kappa (synchronization index). Note that gabazine blanks the effect of UDCA and tauroursodeoxycholate (TUDC) on synchronization. Mineralocorticoid- and glucocorticoid- receptor- antagonists (mifepristone and spironolactone, respectively) did not significantly change effects of UDCA (significance of modulation compared to the control indicated with stars within bars (*: p<0.05). **C.** Examples of neuronal firing patterns recorded from 2 electrodes in one hypothalamic culture (one electrode in black, another in grey color) during 1 second. Note more synchronous discharge of hypothalamic neurons in the presence of UDCA.

Mouse TMN neurons recorded in slices either did not change their activity in response to UDCA 100 µM (n = 5), or significantly reduced their firing compared to the baseline (n = 5, paired t-test, p<0.05). Inhibition of firing showed a delayed onset and represented 86±5% of control at the end of UDCA application (n = 10, Fig. 2A). The firing frequency of cultured mouse hypothalamic neurons on microelectrode arrays (MEA) was not affected by UDCA 10, 30 or 100 µM (Fig. 2B) but UDCA increased the synchronicity of firing within the hypothalamic network (Fig. 2B,C). This effect was abolished in the presence of GABA_A_ receptor antagonist gabazine (Fig. 2B). As conjugated or unconjugated UDCA can interact with the mineralocorticoid- and glucocorticoid- nuclear receptors preventing apoptosis [5], [20] we applied UDCA together with the mifepristone (10 µM, glucocorticoid receptor antagonist) and spironolactone (10 µM, mineralocorticoid receptor antagonist [21]): the UDCA-induced synchronization of firing was not affected (Fig. 2B).

### UDCA decreases amplitude and duration of spontaneous and evoked IPSCs

**Figure 3:**
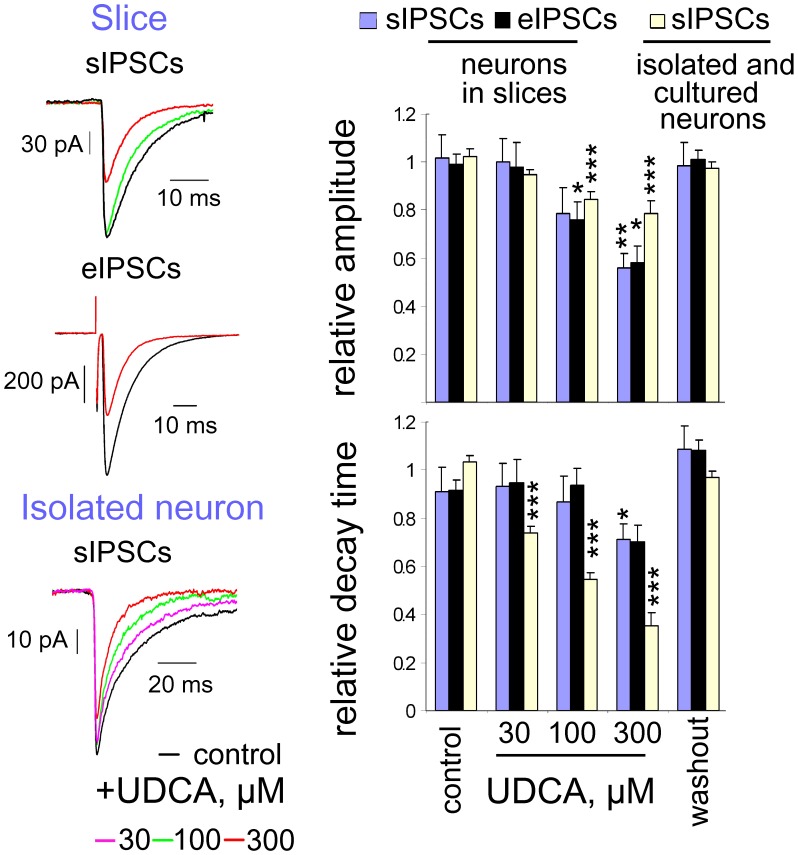
Spontaneous and evoked IPSCs in TMN neurons are inhibited by UDCA. At the left: Examples of averaged (5 min periods) spontaneous and evoked IPSCs recorded from the same neuron in a slice in control (black) and in the presence of UDCA (100 µM: green trace; 300 µM: red trace) and of averaged sIPSCs recorded in an acutely isolated neuron below (each trace represents an average of 24 to 87 events). At the right: Comparison of the relative amplitudes and decay time constants of eIPSCs and sIPSCs from different neuronal preparations. Four to ten neurons were tested with each UDCA concentration. Data obtained from the 4 acutely isolated neurons were pooled with data from 6 cultured neurons. Data points were normalized on the averaged value from control and washout. Significance of difference from control is indicated above the bars (p<0.05; **:p<0.01; ***: p<0.005).

Amplitude and decay time of spontaneous and evoked inhibitory postsynaptic potentials were reduced in the presence of 100 and 300 µM of UDCA in rat TMN neurons (Fig. 3). Rat brain slices were used in these experiments because neuronal identification and stimulation are much more reliable than in the mouse [22]. Evoked IPSCs (eIPSCs) were obtained by stimulating the dense bundle of GABAergic axons entering TMN from VLPO (as in [22]). Gabazine (10 µM) abolished eIPSCs and sIPSCs recorded in these experiments (n = 5, Figure S1). The comparatively slow onset of the UDCA-effects in brain slices can be explained by the poor diffusion of this compound through the slice. Only at 300 µM UDCA produced significant reductions of amplitude and decay time of eIPSCs and sIPSCs in all investigated neurons (analysed with the Kolmogorov-Smirnov 2 sample test in every cell). At 100 µM UDCA significantly inhibited the amplitude of eIPSCs and sIPSCs in less than half of the neurons, 30 µM UDCA was ineffective. In TMN neurons isolated from the same rat hypothalamic slices UDCA inhibited macroscopic GABA-evoked currents with an IC_50_ similar to that seen in mice (Table S1). In TMN neurons recorded from adult HDC−/− mice GABA potency (EC_50_ = 15.6±5 µM, n = 15) and UDCA inhibition (IC_50_ = 90±5 µM, n = 5) of maximal GABA (0.5 mM) responses were not different from WT mice (Figure S2 and Table S1).

Spontaneous IPSCs recorded from isolated neurons were more sensitive to UDCA than sIPSCs recorded in slices (Fig. 3B, C), with UDCA 30 µM significantly reducing decay kinetics in all investigated neurons (n = 4). To exclude effects of the cellular damage during neuronal dissociation, we investigated the block of GABA_A_R by UDCA in mouse neuronal cultures too: UDCA-sensitivity of sIPSCs was indistinguishable in these two preparations (Fig. 3, right plots). UDCA at upto 1.2 mM did not affect AMPA-receptor mediated currents (responses to 150 µM of kainate) and started to block NMDA receptors at concentrations higher than 100 µM (IC_50_ = 540±40 µM, n = 4).

### Expression of GABA_A_ receptors in TMN of histamine deficient mice is not different from wild type littermates

The different modulation of vigilance stages by UDCA in histamine deficient mice compared to their wild type littermates raised the question whether GABA_A_R expression is changed in the absence of histamine. Real-time RT-PCR analysis of GABA_A_R expression in the TMN region revealed no significant difference in mRNA levels encoding for the α1, α2, α5, β1 and γ2 subunits in HDC+/+ (n = 7) versus HDC−/− (n = 6) mice (Figure S2B). The GABA_A_R in HDC−/− TMN neurons tended to be more sensitive to the modulation by vertacetal [23]: EC_50_ = 9.2±3.0 µM (n = 5) in HDC−/− versus 13.4±2.6 µM (n = 5) in wild type neurons (Figure S2C). Although this difference was not significant, increased sensitivity to vertacetal mirrored the slightly higher mRNA level for the β1 subunit of GABA_A_R in HDC−/− mice (2.1±0.7 vs 1.3±0.2 in HDC+/+), indicating that transcriptional up-regulation- or post-translational modification of β1-containing GABA_A_ receptors mediating the TMN inhibition by VLPO during sleep [15] could contribute to the reduced arousal in histamine-deficient mice. No difference in modulation of GABA_A_R by the benzodiazepine-site agonist zolpidem was found between HDC+/+ and HDC−/− TMN neurons (Figure S2D).

### Mechanisms of GABA_A_R block by UDCA

**Figure 4:**
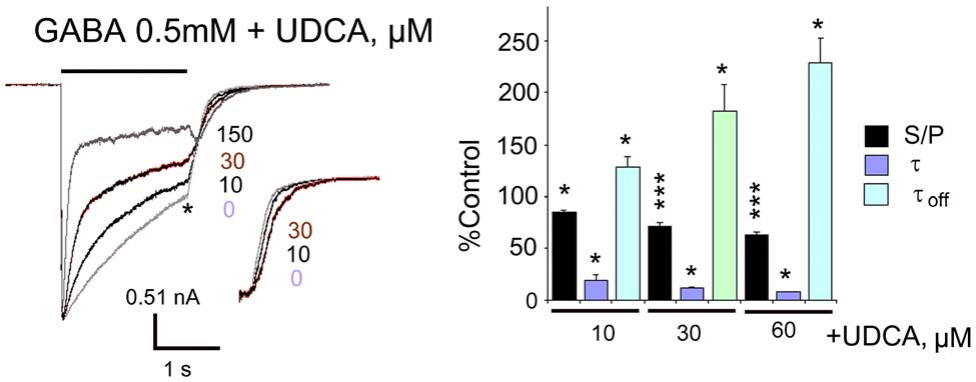
Kinetics of GABA_A_ receptor inhibition by UDCA in TMN neurons. UDCA affects apparent desensitisation of maximal GABA-mediated whole-cell currents. Termination of GABA+UDCA (150 µM) application displays tail current, not obvious at smaller UDCA concentrations, which prolong the decay of GABA-current (τ_off_) after agonist and drug removal (inset shows relaxation currents scaled to the control amplitude (*)). Note, that the peak current amplitude is not affected by UDCA. The bar graph at right shows the summary of UDCA effects on rate and degree of desensitisation obtained from 4–8 neurons. The ratio of steady-state versus peak current (S/P), fast decay time constant (τ) and current decay time constant after removal of drugs (τ_off_) relative to the control values are given. ** p<0.01; ***p<0.005, Wilcoxon test.

**Figure 5:**
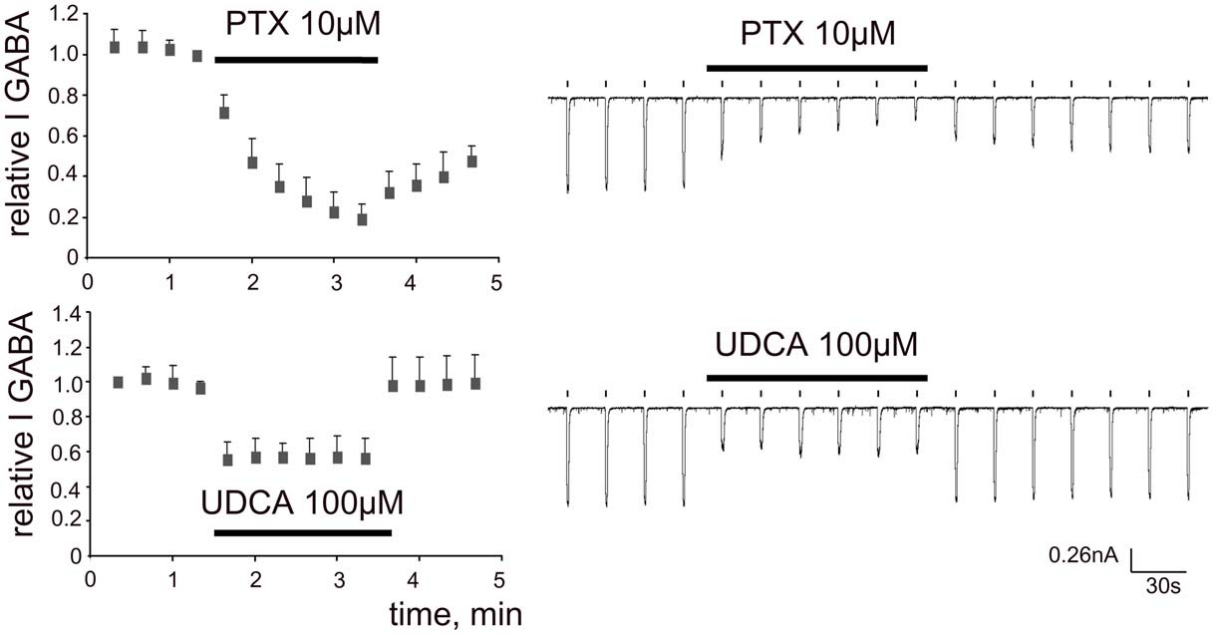
Time courses of GABA-response-block by UDCA and picrotoxin. Picrotoxin (PTX) continuously present in the recording solution shows use-dependent block of GABA (10 µM, 1 s) - currents: block is not fully reversible after PTX withdrawal. In contrast, the UDCA-block is fully reversible and achieves maximum with the first GABA/UDCA co-application.

Similar to chenodeoxycholate [12], UDCA concentration-dependently enhanced macroscopic apparent desensitization of current responses to saturating GABA concentration by decreasing the fast exponential decay time constant and the ratio of steady-state current to peak current. The deactivation time constant (τ_off_) was prolonged by UDCA (Fig. 4). In contrast to the block by picrotoxin, the UDCA-mediated inhibition of GABA currents (taken near EC_50_) was not use dependent (Fig. 5). To determine the voltage dependence of BS antagonism, we investigated the block of the response to GABA at different membrane potentials (Figure S3). The GABA current-voltage relationship was roughly linear from −70 to +30 mV (reversal potential 0.7±2 mV, n = 5, close to the predicted reversal potential for chloride ions: −2.9 mV), but exhibited an outward rectification at more positive potentials (at +50 mV 153±20% of control (−50 mV)). In the presence of UDCA the current-voltage relationship was nearly linear with a slight inward rectification. At +50 mV the current amplitude was 69±5% of control (−50 mV) without a significant change in the reversal potential (3.1±2.2 mV, p = 0.14). Thus, the inhibition of GABA-induced currents was greatest at the most positive holding potentials. In the presence of UDCA, stationary GABA current represented 59±2% and 28±2% of corresponding unblocked currents at −50 and at +50 mV, respectively (the difference in the amount of block at these two membrane potentials was significant, p = 0.012). At positive holding potentials co-application of GABA and UDCA was associated with the appearance of a tail current following cessation of the application. According to the Woodhull model, two sets of GABA currents (steady-state currents in the absence and presence of UDCA) measured at different membrane potentials were organized in a (I_blocked_/I_control_)/Voltage plot and fitted with the equation (3). The results of fitting and experimental data are shown in Figure S3. The best fitted values for the fraction of the transmembrane field sensed at the acceptor site (f) was 0.29±0.03 (n = 5), which did not differ significantly from cholate, chenodeoxycholate and dehydrocholate, indicating the same mechanism of their interaction with the GABA_A_ receptor.

### Minimal structural demands for the BS-mediated inhibition of recombinant GABA_A_Rs

**Figure 6:**
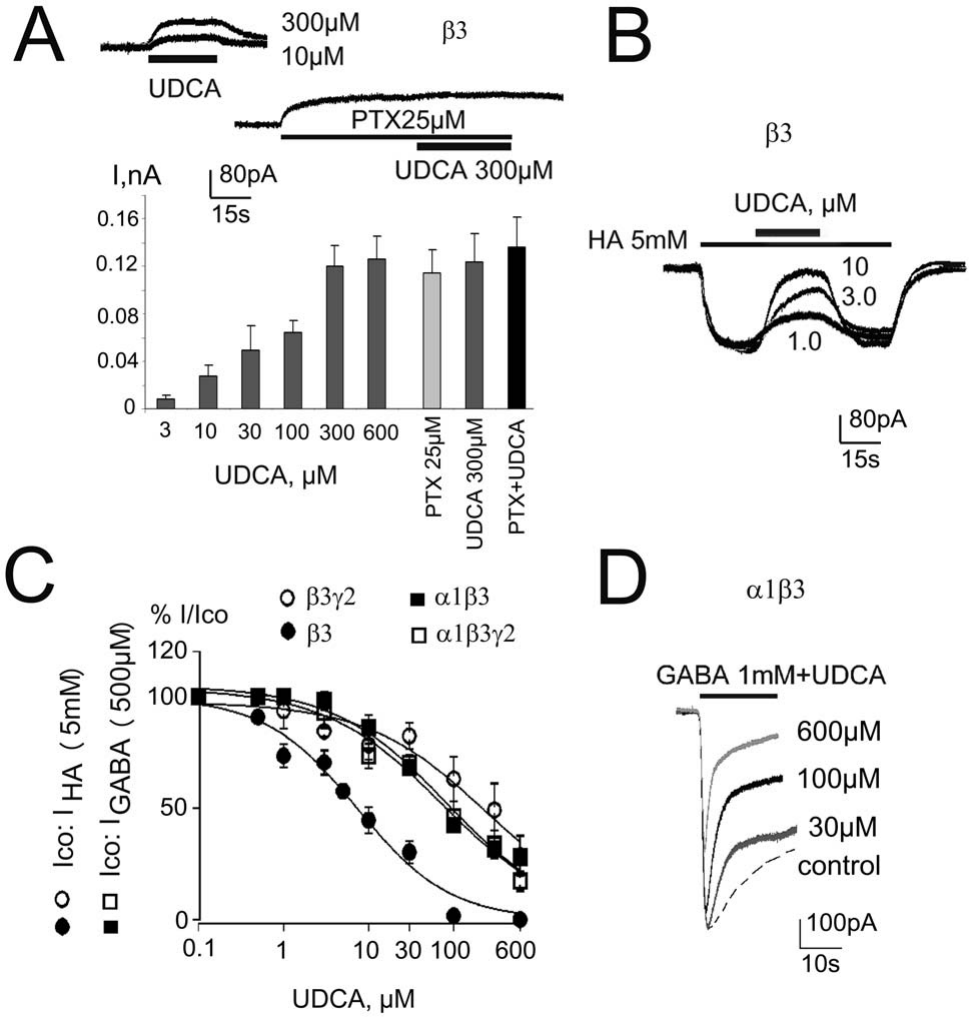
Recombinant α1β3γ2L GABA_A_Rs are blocked by UDCA in a way comparable to native receptors. **A.** UDCA and picrotoxin (PTX) induce outward shift in baseline current in HEK293 cells transfected with β-plasmid (block of constitutively open channels). Plot below shows averaged amplitudes of UDCA- and PTX- responses (4–10 cells for each concentration). Note lack of additivity between maximal UDCA and PTX induced outward currents. **B.** Histamine (HA)-evoked inward currents and their reversible blockade by different concentrations of UDCA at homopentameric GABA_A_ receptors. **C.** Concentration-response curves for UDCA-block of control (co) either GABA (0.5 mM)- or histamine (HA, 5 mM)- evoked currents obtained from 4 different receptor types fitted with the following IC_50_s(n_H_): α1β3γ2L: 73±16 µM (0.63); α1β: 81±16 µM (0.65); β3γ2L: 232±66 µM (0.62); β3: 7.4±2 µM (0.8). Four to ten cells were investigated with the whole concentration range for each receptor type. **D.** Examples of current recordings in α1β3-expressing HEK293 cell: GABA (dotted line)- or GABA+UDCA – responses are superimposed.

Among the 19 known GABA_A_R subunits only a restricted number forms functional combinations in the brain [24]. Our aims were: i) to compare the effects of BS on native and recombinant GABA_A_R of similar subunit composition as seen in hypothalamic neurons (express α1, α2, β1, β3, γ1 and γ2 subunits [23], [25]); ii) to find the minimal structural requirements for the UDCA inhibition. Coapplication of UDCA with 1 mM GABA inhibited the peak current and increased the apparent desensitization rate in cells expressing α1β3 and α1β3γ2L GABA_A_Rs (Fig. 6). The concentrations producing half-maximal effects on steady-state currents (81 µM for α1β3 and 73 µM for α1β3γ2L) were similar to those measured on native receptors (92 µM, Table S1). Changing the subtype of the β subunit had no significant effect on the ability of UDCA to inhibit the currents. The IC_50_ for UDCA was 96±17 µM and 85±11 µM in α1β1γ2L and α1β2γ2L receptors, respectively.

We also examined the action of BS at β3 homopentameric receptors, which undergo spontaneous openings readily detected by using the channel blocker picrotoxin. UDCA induced a concentration-dependent outward shift of baseline current with the half-maximal effects achieved at ∼100 µM. In accordance with the same target (GABA_A_ receptor) the amplitudes of outward currents in response to maximal effective UDCA (300, 600 µM) and picrotoxin (25 µM) were not additive (Fig. 6A).

As GABA does not reliably activate β3 and β3γ2L receptors, histamine [26] was used as a ligand of these receptors whereas at other receptor types maximal GABA-evoked currents were investigated. At homopentameric β3 receptors histamine (5 mM) - evoked currents were inhibited by UDCA with an IC_50_ = 7.4 µM. At all other receptor types UDCA was a less potent modulator of GABA_A_ receptor gating (Fig. 6 B,C).

### GABA_A_R inhibition by BS is reduced by the α1V256S mutation

**Figure 7:**
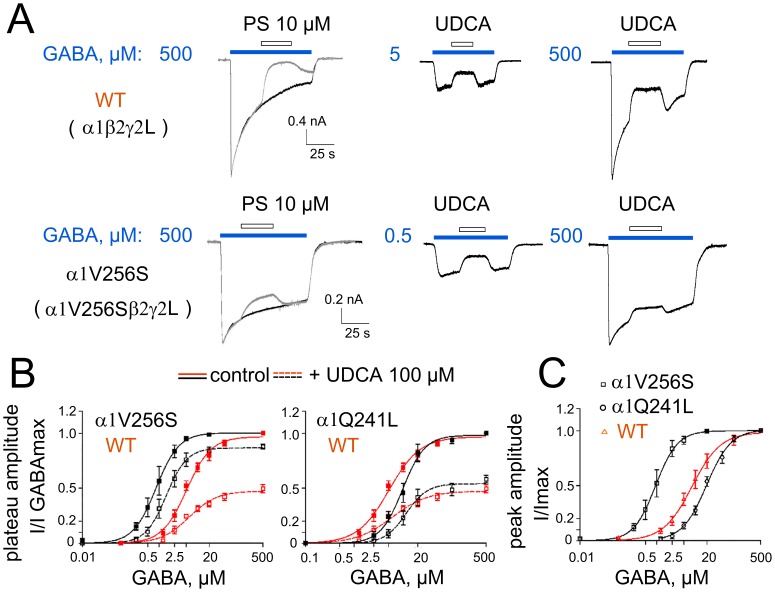
Impairment of GABA_A_R-block by UDCA in the mutant α1V256S- but not α1Q241L-containing receptors. **A.** GABA-evoked currents, in response to maximal concentrations and concentrations around EC_25_, and their inhibition by pregnenolone sulphate (PS) or UDCA 100 µM. **B.** GABA dose-response curves constructed for the plateau amplitudes measured for control: immediately before UDCA application, which started 15–25 s after the beginning of the GABA application; and for the amplitude of blocked current: at the beginning of UDCA application (first point at a steady-state level). The inhibition of maximal GABA-responses by UDCA is significantly smaller in WT (47±3% of control, n = 7) and mutant α1Q241L receptors (54±2% of control, n = 5, no difference with WT) than in the mutant α1V256S receptors (87±1% of control, n = 5; p = 0.0047 vs WT). UDCA does not significantly modify the EC_50_ and n_H_ values for GABA in the WT (6±1 µM; 1.2±0.2 versus 5.1±0.5 µM; 1.4±0.1 in control), in the mutant α1Q241L (11.5±1.2 µM; 2.0±0.4 versus 9.8±0.7 µM; 1.9±0.2 in control) and α1V256S (1.5±0.07 µM; 1.6±0.1 versus 0.83±0.03 µM; 1.5±0.06 in control) receptors. **C.** GABA dose-response curves constructed from peak current amplitude values normalized on maximal GABA-evoked current for the WT (EC_50_ = 8±0.5 µM; n_H_ = 1.2±0.1; n = 8), α1V256S (EC_50_ = 0.9±0.03 µM; n_H_ = 1.5±0.06; n = 5) and α1Q241L (EC_50_ = 20±1 µM; n_H_ = 1.4±0.1; n = 5) receptors of α1β2γ2L-composition expressed in HEK293 cells.

The actions of BS on native and recombinant GABA_A_ receptors expressed in HEK 293 cells appear kinetically similar to those observed in the presence of inhibitory neurosteroids, e.g., pregnenolone sulphate, which enhances the apparent rate of desensitization and, under some experimental conditions, reduces the peak amplitude [27]. A previous study showed that a mutation to the 2′residue in the α-subunit M2 transmembrane domain (α1V256S) strongly reduces inhibition by neurosteroids [28]. Here, we tested the effect of the α1V256S mutation on GABA_A_R inhibition by UDCA. In wild-type receptors, the UDCA-inhibition did not depend on the GABA concentration (Fig. 7A): at all GABA concentrations tested UDCA (100 µM) inhibited control GABA responses to the same extent. The relative amplitude of maximal GABA-responses was reduced by UDCA in the mutant α1V256S receptors to 87±1% of control (n = 5), differing significantly from wild type receptors (47±3% of control, n = 7, p<0.005, Fig. 7A, B). Pregnenolone sulphate (10 µM) inhibited the steady-state amplitude of maximal GABA-currents to 11±3% of control in WT and to 82±6% of control in mutated α1V256Sβ2γ2L receptors (p<0.001)(Fig. 7A). UDCA's blocking potency was significantly reduced (p<0.01) in the mutant α1V256S receptors (IC_50_ = 330±41 µM (n = 4) vs 84.7±11.3 µM (n = 5) in WT). UDCA did not significantly modify the GABA EC_50_ and n_H_ values in the WT and α1V256S receptors (Fig. 7B).

We also tested the involvement of the site mediating the action of potentiating neurosteroids in BS-modulation. The α1Q241L mutation has been shown to abolish GABA_A_R potentiation by several neurosteroids and analogues [29]. Our data demonstrate that the α1Q241L mutation has no effect on GABA_A_R inhibition by UDCA. The presence of 100 µM UDCA reduced the maximal steady-state current elicited by 500 µM GABA from cells expressing the α1Q241L mutant receptor to 54±2% of control (n = 5 cells, Fig. 7B). This is similar to the inhibition produced by UDCA in wild-type receptors (p = 0.14). Both types of mutant receptors differed significantly from WT receptors in their sensitivity to GABA (Fig. 7C).

## Discussion

We discovered a wake-promoting action of UDCA in mice at a dose used for the standard treatment of primary biliary cirrhosis. Cortical EEG and sleep-wake monitoring showed a difference between wild type and histamine-deficient mice in response to UDCA, indicating that recruitment of histamine is necessary in the wake-promoting effect. In vitro patch-clamp recordings from histaminergic neurons revealed no increase in firing rate by UDCA, but inhibition of GABAergic currents. Hypothalamic network activity recorded with MEAs was synchronized by UDCA through a reduction of GABAergic inhibition. Synchronization of neuronal firing is expected to increase histamine release from varicosities.The GABA_A_R was blocked by UDCA also in HEK293 cells carrying recombinant receptors of different subunit combinations. We conclude that the UDCA action at the GABA_A_R in the central nervous system and the periphery (lymphocytes, liver, lung and pancreas) takes place at a new specific site.

The wake-promoting action of UDCA is in keeping with GABA_A_R antagonism and the well known sedative effect of GABA_A_R agonists like muscimol applied either systemically or locally into the posterior hypothalamus. In contrast to cholate and chenodeoxycholate (all found in the brain) [12] UDCA did not suppress neuronal activity and did not block NMDA receptors. UDCA and other BS [12] shorten the kinetics of spontaneous GABAergic synaptic potentials with potencies similar to those obtained from macroscopic GABA-evoked currents on acutely isolated histaminergic neurons. Investigation of the mechanisms of GABA_A_R block by BS in our previous study [12] showed rapid BS binding to the open, but not to the closed (unliganded) state of the receptor. No inhibitory action of BS on the closed channel was observed. In order to explore further the possibility of an open channel block we compared the action of UDCA with the action of a classical open channel blocker. In contrast to the picrotoxin-block, the UDCA-block was not use-dependent. In spite of the non-additive actions of UDCA and picrotoxin at homopentameric β3 receptors expressed in HEK293 cells, mutational analysis convincingly demonstrated that the picrotoxin-block involves mechanisms different from those employed by BS. The UDCA- block was impaired in α1β2γ2L receptors containing a mutated α-subunit (α1V256S). This mutation is known to reduce the inhibition by pregnenolone sulphate [27] but it does not affect the potency of picrotoxin [30]. Another mutation at the GABA_A_R α-subunit (α1Q241L) which is known to abolish the potentiating action of neurosteroids [29] had no influence on GABA_A_R inhibition by UDCA. The similarity of GABA_A_R block by UDCA in TMN neurons and in HEK293 cells and dependence of this block on the α1V256 site in the α-subunit thus provided evidence for the direct interaction of BS with the GABA_A_ receptor. Unfortunately the mutation α1V256S causes a dramatic change in receptor sensitivity to GABA (the same is true for the mutation α1Q241L), a situation that excludes the generation of mutant mice carrying these mutations. Therefore it is not possible at present to elucidate whether conditional (HDC/cre driven) expression of the α1V256S mutation in TMN neurons would impair the action of UDCA on waking stages with EEG recordings.

The wake-promoting action of UDCA in wild type but not in HDC knockout mice was evident at 32 mg/kg. This dose is nearly 20 times below the one necessary to combat retinal degeneration in the mouse (500 mg/kg) [2], [31]. We found significant changes in cortical EEG starting from the third hour after oral UDCA application, in keeping with the serum peak time of the UDCA concentration (∼18±10 µM) in volunteers (men) taking a single oral dose of UDCA (∼12 mg/kg) [32]. Previous studies have shown that UDCA reaches the CNS [33]. We also found that UDCA enhances arousal during the active period of the day, an effect that was reversed in histamine-deficient mice. This clearly suggests involvement of the histaminergic system. It is known that histaminergic neurons are spontaneously active during waking and silent during sleep [14] and that GABAergic inputs play a major role in inactivating histaminergic neurons and thus promoting sleep, although the sleep-wake balance is also controlled by potent circadian mechanisms, other excitatory and inhibitory inputs and arousal systems [34]. As UDCA enhanced wakefulness during the active period but not during the sleepy period in wild type mice, it appears as if the GABA_A_R-mediated disinhibition could effectively change the activity of histamine neurons and lead to wake enhancement only when excitatory inputs to histamine neurons are enhanced as a consequence of behavioral activation. When the excitatory inputs decrease during the sleepy period, such a disinhibition alone would be insufficient to impact TMN neuronal firing.

Effects described here on the sleep-wake patterns in wild type mice were missing in mice lacking histamine (HDC−/− mice). Whereas these data clearly demonstrate the histamine-dependent nature of the wake-promoting effect of UCDA, it remains to determine why, intriguingly, UCDA caused opposite effects in HDC KO mice, i.e., decrease in waking and increase in total sleep (SWS+PS). It is possible that, without histamine in TMN neurons, the GABA_A_R-mediated disinhibition would enhance or facilitate the release of other neurotransmitters defined in wild type animals as co-transmitters of histamine: GABA or galanin for instance are inhibitory. We did not detect any significant difference in GABA_A_R expression or function in the posterior hypothalamus of HDC−/− mice. The possible circadian variation in GABAergic systems between wild type and knockout mice also remains to be investigated.

Besides the GABA_A_R other central and peripheral sites of UDCA action cannot be ignored; among those nuclear receptors [5], [20], the BS receptor TGR5 (which is rather insensitive to UDCA [35]), BS transporters and intracellular signal cascades [36].

We show that central histamine is necessary for the wake-promoting action of UDCA. The hypothalamic histamine system is involved in the control of energy balance, endocrine functions and sleep-wake regulation [14]. Ionotropic receptors for histamine (with yet elusive structures) control the excitability of oxytocin neurons in the supraoptic nucleus [37]. One likely candidate is a GABA_A_ receptor devoid of GABA-binding site [26]. We show here that β3 homopentameric GABA_A_ receptors gated by histamine represent the most sensitive target for BS (IC_50_ 7.4 µM). Synaptic GABA_A_Rs (like γ2-subunit-containing recombinant receptors) were less sensitive to UDCA: thus 10 µM of UDCA significantly accelerated the decay kinetics of sIPSCs in some and 30 µM in all TMN neurons. Hypothalamic wake-on neurons, such as histaminergic and orexinergic (hypocretinergic) neurons are under tonic GABAergic inhibition. Local injection in rat of a GABA_A_R antagonist in the perifornical area containing orexin-neurons induces cortical arousal [38]. These and other wake-on neurons expressing UDCA-sensitive GABA_A_ receptors may represent further important sites of UDCA action.

Our study expands the list of extrahepatic BS-targets and suggests that BS may play regulatory roles in the brain and at peripheral sites interacting with the GABA_A_R. This finding is particularly relevant for the development of new therapeutics to treat neurological abnormalities in hepatic encephalopathy, accompanied by an increased GABAergic tone. It was suggested recently that reduced locomotor activity in hyperammonemic rats can be recovered by the GABA_A_R antagonist bicuculline [19]. Rats with liver cirrhosis show decreased wakefulness [39]. The wake-promoting potential of UDCA awaits to be tested in these models of hepatic encephalopathy.

In conclusion, the clinical benefit of UDCA is complemented by its wake-promoting action during the active period of the day. This involves a GABA_A_R-mediated disinhibition of the histaminergic system.

## Materials and Methods

### Polygraphic recording in the mouse and analysis of sleep-wake parameters

Animal experiments were conducted according to the Animal Protection Law of the Federal Republic of Germany and the local guidelines (Bezirksregierung Duesseldorf) and were in accordance with the European Communities Council directive regarding care and use of animals for experimental procedures (86/609/EEC). The general protocol of sleep-wake recording and experiment in the mouse was approved by the ethic committee of Claude Bernard University of Lyon. All efforts were made to minimize the number of animals and their suffering. At the age of 12 weeks and with a body weight of 30±2 g HDC wild type and knockout mice used for EEG and sleep-wake studies were chronically implanted, under deep gas anesthesia using isoflurane (2%, 200 ml/min) and a TEM anesthesia system (Bordeaux, France), with six cortical electrodes (gold-plated tinned copper wire, Ø = 0.4 mm, Filotex, Draveil, France) and three neck muscle electrodes (fluorocarbon-coated gold-plated stainless steel wire, Ø = 0.03 mm, Cooner Wire Chatworth, CA, U.S.A.) to record the electroencephalogram (EEG) and electromyogram (EMG) and to monitor the sleep-wake cycle. Finally, the electrode assembly was anchored and fixed to the skull with Super-Bond (Sun Medical Co., Shiga, Japan) and dental cement. This implantation allows stable and long-lasting polygraphic recordings.

After surgery, the animals were housed individually in barrels placed in an insulated sound-proof recording room maintained at an ambient temperature of 22±1°C and on a 12 h light/dark cycle (lights-on at 7 a.m.), standard food and water being available ad libitum. After a 7-days recovery period, mice were habituated to the recording cable for 7 days before polygraphic recordings were started. Cortical EEG (contralateral frontoparietal leads) and EMG signals were amplified, digitized with a resolution of 256 and 128 Hz, respectively, and computed on a CED 1401 Plus (Cambridge, UK). Using a Spike2 script and with the assistance of spectral analysis using the fast Fourier transform, polygraphic records were visually scored by 30-sec epochs for wakefulness (W), slow wave sleep (SWS), and paradoxical sleep (PS) according to previously described criteria validated for mice [40].

Animals were subjected to 12 h sleep-wake recordings following administration of URSOFALK (clinically used UDCA, 32 mg/kg) through the oral route (per os) at 10 a.m. (during the sleepy period) or at 7 p.m. (during the active period). Each animal received twice single dose of UDCA and twice equal volume of vehicle. There was an interval of 7 days between any two administrations. UDCA or vehicle was applied at random. The waking, slow wave sleep or paradoxical sleep amounts were compared between vehicle and UCDA within the same animal, the differences being averaged within the groups. Statistical analysis was performed with Dunnett's t test and ANOVA for repeated measures. Significance level was set at p<0.05. Data are presented as mean ± standard error of the mean (SEM).

### Electrophysiology in native neurons, slices from rat hypothalamus

Recordings from histaminergic neurons in male Wistar rat (22–26 day old) or 30–60 day old mouse (SV129, histidine decarboxylase deficient HDC−/− or their wild type littermates) hypothalamic slices were performed as previously described [16]. Slices were obtained between 9 and 11 a.m. In the beginning of whole-cell voltage clamp recordings TMN neurons were identified with the help of DIC microscopy and electrophysiological criteria as previously described [40]. Inhibitory postsynaptic currents (IPSCs) were evoked by local stimulation through a bipolar stainless-steel stimulating electrode (50 µm; WPI, UK). The stimulation of axonal endings coming to TMN from ventrolateral preoptic area (VLPO) was done as described in [22]. Visual identification of histaminergic neurons recorded in cell-attached configuration (see [16]) was confirmed by the significant reduction of firing rate by the histamine 3 (H3) receptor agonist R-α-methylhistamine (0.2 µM), which was performed at the end of each experiment. Individual neurons were separated by vibrodissociation after brief preincubation with papain from the hypothalamic slices of mice or rats and identified as TMN neurons according to their size and shape. Identification was confirmed post hoc with the single-cell RT-PCR (all data presented in the manuscript were obtained from histidine decarboxylase-expressing neurons) [23], [40]. TMN neurons from HDC−/− mice were identified by the expression of peripherin [16]. Whole-cell voltage-clamp recordings from isolated neurons were done with an EPC-9 amplifier and TIDA for Windows (HEKA, Lambrecht, Germany) at room temperature. Membrane potentials were not corrected for liquid junction potential (5.6 mV, calculated with LJPCalc, Barry, 1994). The intracellular solution was composed of (in mM) 140 KCl, 2 MgCl_2_, 0.5 CaCl_2_, 5 EGTA, 2 ATP and 10 HEPES (pH 7.2 adjusted with NaOH). A fast perfusion technique was used for application of ligands and modulators of fast ionotropic receptors [25], [41]. UDCA was co-applied with GABA, NMDA or kainate, see [12]. Concentration-response relationships for GABA was fit with the following equation (1): *R* = *R*_max_/(1+(EC_50_/[ligand])^n^); where *R*_max_ is the relative maximal response elicited by the ligand. Agonist-mediated responses were normalized to the 500 µM- GABA. EC_50_ is the ligand concentration producing a half-maximal response, [ligand] is ligand concentration, and n is the Hill slope. Hill coefficients were obtained from the slope of the log/log plot of the dose-response curves. The inhibition curves of GABA -evoked currents by UDCA were fitted with the following equation (2): *R* = *R*max/(1+(IC_50_/[L])^n^); where *R*max is the maximal degree of block of the agonist-mediated response achieved by the tested blocker, IC_50_ is the concentration of the blocker producing a half-maximal block of agonist-mediated responses, [L] is the blocker concentration and n is the Hill coefficient. Steady-state amplitudes of the responses were used for the construction of dose-response plots if not mentioned otherwise.

For the testing of UDCA action at NMDA receptors a maximal 0.2 mM concentration of agonist was used. Recordings were done in Mg^2+^ -free solution in the presence of 10 µM of glycine. NMDA-currents were abolished by the NMDA receptor antagonist D-AP5 (100 µM).

Voltage dependence of GABA_A_R block by UDCA was analysed according to the method of Woodhull [42], which provides a means of calculating the fraction of the transmembrane field sensed by charged blocking ligand at its acceptor site. Two sets of GABA currents (steady-state currents in the absence and presence of BS) were measured in the same cell at different membrane potentials. The relationship between block (B = I_blocked_/I_control_) and membrane potential (V) was approximated by equation (3): *B* = 1/(1+([BS]/*K_D_*(0))*exp(f*FV*/*RT*)); where *K_D_*(0) is the dissociation constant of the BS binding site on the GABA_A_R complex at a transmembrane potential of 0 mV, f is the fraction of the electric field that would be transversed by a single charge to produce the observed voltage dependence, *F* is the Faraday constant, R is the universal gas constant and T is the ambient absolute temperature [*F*/(*RT*) = 0.03972 mV^−1^], [BS]-bile salt concentration (mM).

### Microelectrode array (MEA) recordings

Primary dissociated cultures of posterior hypothalamus were prepared from newborn mice according to the protocols previously described [25], [43]. Dissociated cells were plated at a density of 1 to 2×10^5^/cm^2^ onto polyethylenimine -coated MEAs in a volume of 100 µl (microelectrode arrays, Multi Channel Systems, Reutlingen, Germany) or on coverslips (for patch-clamp recordings) and cultured in an incubator with 5% CO_2_, 95% air and 98% relative humidity, at 35.5±0.5°C. On the second day serum-free neurobasal medium containing supplement B27 (2%) was added to the final volume of 1 ml. Recordings were done between 11th–21st day after plating. Extracellular potentials were recorded on MEAs with a square grid of 60 planar Ti/TiN-microelectrodes (30 µm diameter, 200 µm spacing) at 37°C. Signals from all 60 electrodes were simultaneously sampled at 25 kHz, visualized and stored using the standard software MCRack provided by Multi Channel Systems. Spike detection and analysis was performed offline with the software SpAnNer (RESULT Medizinische Analyseverfahren, Tönisvorst, Germany), which uses the agreement coefficient “Cohen's kappa” (κ) to assess the degree of spike and burst synchrony for selected pairs of electrodes. Theoretically, kappa may vary in the range from −1 to +1. −1 is obtained for zero coincident events, while +1 is obtained for maximal coincidence. In order to describe the distribution of kappa across the whole MEA, we calculated the mean of all kappa-values obtained for pairs of “active” electrodes.

At the beginning of experiments the basal medium was replaced by a magnesium-free HEPES-based recording solution (see above) and measurements were started after a 20 min adaptation phase. Every measurement comprised three recordings – control, test substance and washout (second control) – each two minutes long and separated by an intermediate period of 30 seconds. Recordings were made from MEAs if more than 10 channels were active.

### HEK293 cell line maintenance, transfection with the GABA_A_R plasmids and patch-clamp recordings

HEK 293 cells were obtained from DSM (Deutsche Sammlung von Mikroorganismen und Zellkulturen GmbH, DSM, Braunschweig). They were grown and maintained according to published standard protocols [26]. Briefly, cells were grown in minimal essential medium supplemented with 10% fetal bovine serum, 100units/ml penicillin and streptomycin and 2 mM L-glutamine and splitted once per week. Semiconfluent cells were transfected on the third day after splitting with the help of Lipofectamine 2000 (Invitrogen), recordings were done 48–72 hrs after transfection.

The GABA_A_R subunit cDNAs, generously donated by G.Gisselmann (rat α1, mouse γ2L, rat β1, β2 and human β3 [26]) and J.H. Steinbach (rat α1V256S [28] and rat α1Q241L [44]), were subcloned into pcDNA3 (Invitrogen, Karlsruhe, Germany) and applied in a ratio 1∶1∶1 for the α1βxγ2L receptor and 1∶1 for α1β3 or β3γ2L receptors. Integration of the γ-subunit was controlled by potentiation of the control GABA response (near EC_10_) by zolpidem (1 µM) and by the resistance of the GABA response (near EC_50_) to ZnCl_2_ (10 µM). For each receptor type patch clamp recordings were obtained from at least three separate transfections. Whole-cell patch clamp recordings were done in a similar extracellular solution as for the native cells (see above) except that the concentration of MgCl_2_ was reduced to 1.2 mM. Intracellular electrodes contained 140 mM CsCl (instead of KCl), pH adjusted with CsOH. The voltage was held at −40 mV.

All chemicals used for the in vitro experiments were purchased from Sigma-Aldrich (Taufkirchen, Germany). Drugs were diluted and stored as recommended. Statistical analysis was performed with the non-parametrical Mann-Whitney U-test if not indicated otherwise. Significance level was set at p<0.05. Data are presented as mean ± standard error of the mean (SEM).

## Supporting Information

Figure S1 The GABA_A_ receptor antagonist gabazine (gz) blocks spontaneous and evoked inhibitory postsynaptic currents (sIPSCs and eIPSC, respectively). A. Averaged eIPSCs recorded during 5 min in control and in the presence of gabazine. B. Representative traces of slice recording show evoked (after stimulus artefact, marked with arrow) and spontaneous IPSCs. C. GABAergic sIPSCs recorded in isolated TMN neuron, abolished by gabazine.
(DOC)

Figure S2 Comparison of GABA_A_ receptor functional expression in histamine deficient mice (HDC−/−) and their wild type littermates (HDC+/+). A. Responses to different GABA concentrations in two TMN neurons, identified with single-cell RT-PCR by the expression of peripherin. B. Real-time RT-PCR analysis of GABA_A_ receptor expression in TMN region of 7 wild type and 6 knockout mice. No significant difference was found. C. Modulatory potency of vertacetal does not differ between wild type and knockout mice. For the calculation of relative potentiation in each recorded neuron, the amplitude of the control response (GABA at concentration ∼EC_15_) was subtracted from all amplitudes in the presence of vertacetal. All “potentiation” values were normalized on maximal potentiation. D. Zolpidem (zp) potentiation obtained in TMN neurons from wild type and HDC knockout mice. Averaged dose-response curves for zolpidem potentiation are shown at the right. Number of investigated neurons in brackets.
(DOC)

Figure S3 Voltage dependence of UDCA block. A.Example of GABA-evoked currents recorded at holding potentials −70, −50, −30, −10, 10, 30, 50 and 70 mV in control (left) and in the presence of UDCA (right). Stars indicate the time point where amplitude was measured for the construction of I/V plots. Holding potential was adjusted 10 s before GABA exposures during time indicated by lines above the traces. B. Current-voltage relationships for control currents and currents blocked by UDCA. I–V curves were constructed from measurements in 5 cells. All responses obtained in one cell were normalized to the response at −50 mV (arrow). C. Woodhull analysis of BS block. GABA_A_R block by several common BS and UDCA is shown for comparison. Normalized block is plotted versus holding potential. The data are fitted using Eq.3. The best fitted values for KD(0) and f were 42 µM and 0.33±0.02 (n = 4, CDOC, in red); 64 µM and 0.29±0.03 (n = 5, UDCA, in black); 0.71 mM and 0.3±0.02 (n = 5, cholate, in green); 1.4 mM and 0.33±0.03 (n = 3, dehydrocholate, in pink).
(DOC)

Table S1 A comparison of BS potencies at native GABA_A_ receptors versus recombinant receptors of different compositions expressed in HEK 293 cells. Recombinant GABA_A_ receptors were activated with the maximal GABA concentration (50–1000 µM) except (*) for the β3 homopentameric receptors, where amplitudes of outward currents, evoked by UDCA application, were analysed. As multiple applications of UDCA in these experiments caused run-down of block of spontaneous channel openings, IC_50_ is roughly estimated (∼). All IC_50_s values were compared with those obtained for the juvenile mouse neurons. Only in case of the mutated α1 (V256S) subunit the potency of UDCA was significantly different.
(DOC)
