# Supplementary material for: Waking Action of Ursodeoxycholic Acid (UDCA) Involves Histamine and GABAA Receptor Block
Source: PLoS One. 2012 Aug 6;7(8):e42512. doi: 10.1371/journal.pone.0042512 (PMC3412845; doi:10.1371/journal.pone.0042512)
Supplement: Figure S1 — The GABAA receptor antagonist gabazine (gz) blocks spontaneous and evoked inhibitory postsynaptic currents (sIPSCs and eIPSC, respectively). A. Averaged eIPSCs recorded during 5 min in control and in the presence of gabazine. B. Representative traces of slice recording show evoked (after stimulus artefact, marked with arrow) and spontaneous IPSCs. C. GABAergic sIPSCs recorded in isolated TMN neuron, abolished by gabazine. (DOC) [file pone.0042512.s001.doc]

**
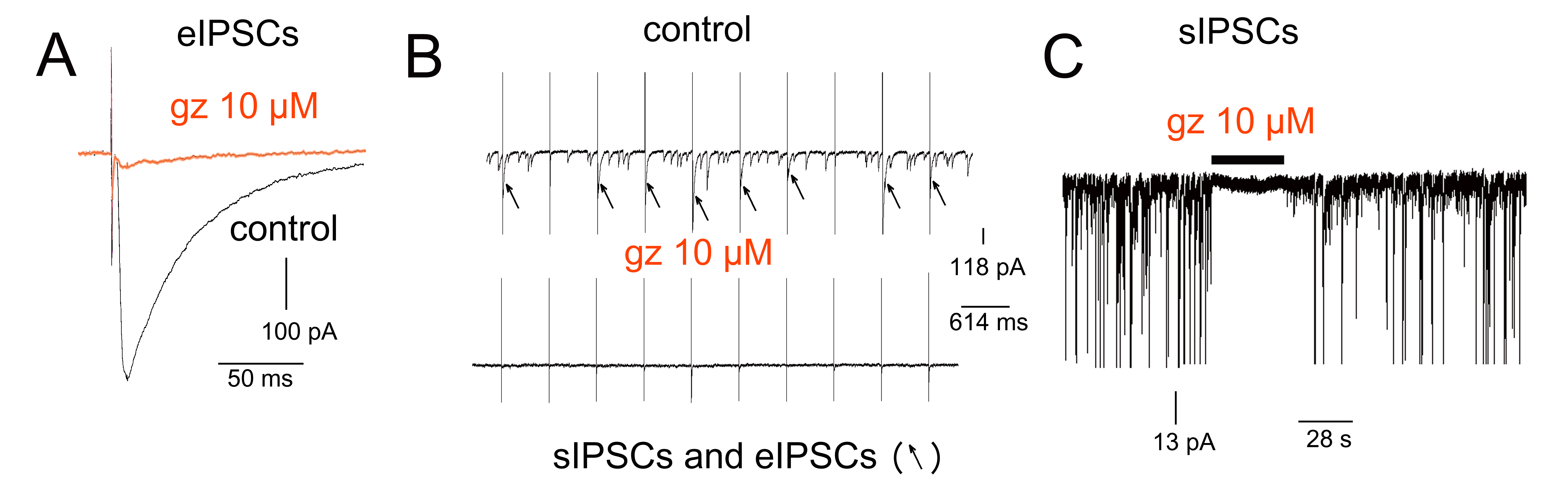
**

**Supplementary Figure 1.** The GABAA receptor antagonist gabazine (gz) blocks spontaneous and evoked inhibitory postsynaptic currents (sIPSCs and eIPSC, respectively). A. Averaged eIPSCs recorded during 5min in control and in the presence of gabazine. B. Representative traces of slice recording show evoked (after stimulus artefact, marked with arrow) and spontaneous IPSCs. C. Gabaergic sIPSCs recorded in isolated TMN neuron, abolished by gabazine.
