# Supplementary material for: Waking Action of Ursodeoxycholic Acid (UDCA) Involves Histamine and GABAA Receptor Block
Source: PLoS One. 2012 Aug 6;7(8):e42512. doi: 10.1371/journal.pone.0042512 (PMC3412845; doi:10.1371/journal.pone.0042512)
Supplement: Figure S2 — Comparison of GABAA receptor functional expression in histamine deficient mice (HDC−/−) and their wild type littermates (HDC+/+). A. Responses to different GABA concentrations in two TMN neurons, identified with single-cell RT-PCR by the expression of peripherin. B. Real-time RT-PCR analysis of GABAA receptor expression in TMN region of 7 wild type and 6 knockout mice. No significant difference was found. C. Modulatory potency of vertacetal does not differ between wild type and knockout mice. For the calculation of relative potentiation in each recorded neuron, the amplitude of the control response (GABA at concentration ∼EC15) was subtracted from all amplitudes in the presence of vertacetal. All “potentiation” values were normalized on maximal potentiation. D. Zolpidem (zp) potentiation obtained in TMN neurons from wild type and HDC knockout mice. Averaged dose-response curves for zolpidem potentiation are shown at the right. Number of investigated neurons in brackets. (DOC) [file pone.0042512.s002.doc]

**

**

**Supplementary Figure 2. Comparison of GABA_A_ receptor functional expression in histamine deficient mice (HDC-/-) and their wild type littermates (HDC+/+).** **A**. Responses to different GABA concentrations in two TMN neurons, identified with single-cell RT-PCR by the expression of peripherin. **B**. Real-time RT-PCR analysis of GABA_A_ receptor expression in TMN region of 7 wild type and 6 knockout mice. No significant difference was found. **C.** Modulatory potency of vertacetal does not differ between wild type and knockout mice. For the calculation of relative potentiation in each recorded neuron, the amplitude of the control response (GABA at concentration ~EC_15_) was subtracted from all amplitudes in the presence of vertacetal. All “potentiation” values were normalized on maximal potentiation. **D**. Zolpidem (zp) potentiation obtained in TMN neurons from wild type and HDC knockout mice. Averaged dose-response curves for zolpidem potentiation are shown at the right. Number of investigated neurons in brackets.
