# Supplementary material for: Waking Action of Ursodeoxycholic Acid (UDCA) Involves Histamine and GABAA Receptor Block
Source: PLoS One. 2012 Aug 6;7(8):e42512. doi: 10.1371/journal.pone.0042512 (PMC3412845; doi:10.1371/journal.pone.0042512)
Supplement: Figure S3 — Voltage dependence of UDCA block. A.Example of GABA-evoked currents recorded at holding potentials −70, −50, −30, −10, 10, 30, 50 and 70 mV in control (left) and in the presence of UDCA (right). Stars indicate the time point where amplitude was measured for the construction of I/V plots. Holding potential was adjusted 10 s before GABA exposures during time indicated by lines above the traces. B. Current-voltage relationships for control currents and currents blocked by UDCA. I–V curves were constructed from measurements in 5 cells. All responses obtained in one cell were normalized to the response at −50 mV (arrow). C. Woodhull analysis of BS block. GABAAR block by several common BS and UDCA is shown for comparison. Normalized block is plotted versus holding potential. The data are fitted using Eq.3. The best fitted values for KD(0) and f were 42 µM and 0.33±0.02 (n = 4, CDOC, in red); 64 µM and 0.29±0.03 (n = 5, UDCA, in black); 0.71 mM and 0.3±0.02 (n = 5, cholate, in green); 1.4 mM and 0.33±0.03 (n = 3, dehydrocholate, in pink). (DOC) [file pone.0042512.s003.doc]

**
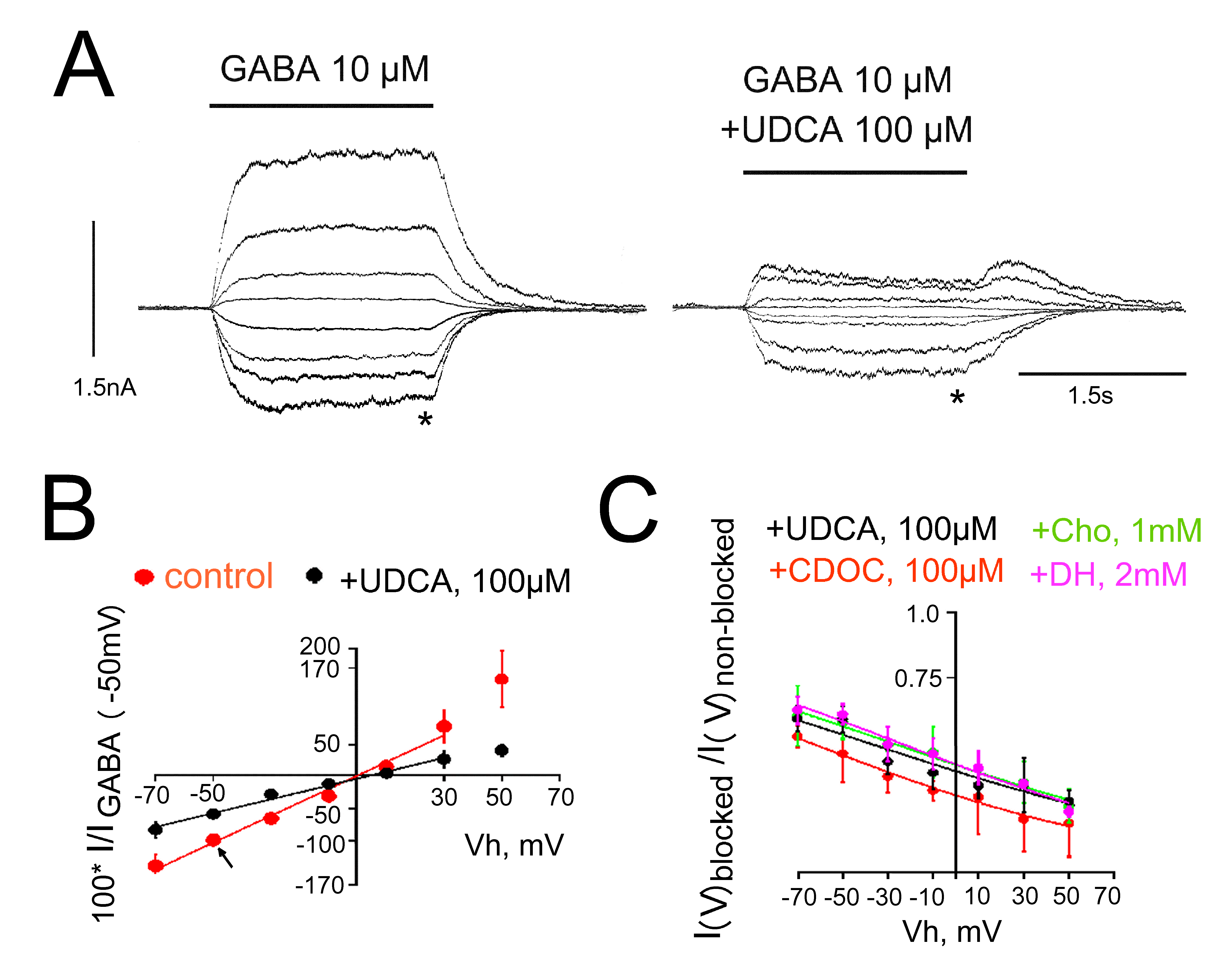
**

**Supplementary Figure 3. Voltage dependence of UDCA block.** **A**.Example of GABA-evoked currents recorded at holding potentials -70, -50, -30, -10, 10, 30, 50 and 70mV in control (left) and in the presence of UDCA (right). Stars indicate time point where amplitude was measured for the construction of I/V plots. Holding potential was adjusted 10s before GABA exposures during time indicated by lines above the traces. **B.** Current-voltage relationships for control currents and currents blocked by UDCA. I-V curves were constructed from measurements in 5 cells. All responses obtained in one cell were normalized to the response at -50mV (arrow). **C.** Woodhull analysis of BS block. GABA_A_R block by several common BS and UDCA is shown for comparison. Normalized block is plotted versus holding potential. The data are fitted using Eq.3. The best fitted values for KD(0) and f were 42 µM and 0.33 ± 0.02 (n=4, CDOC, in red); 64 µM and 0.29 ± 0.03 (n=5, UDCA, in black); 0.71 mM and 0.3 ± 0.02 (n=5, cholate, in green); 1.4 mM and 0.33 ± 0.03 (n=3, dehydrocholate, in pink).
