# Supplementary material for: Waking Action of Ursodeoxycholic Acid (UDCA) Involves Histamine and GABAA Receptor Block
Source: PLoS One. 2012 Aug 6;7(8):e42512. doi: 10.1371/journal.pone.0042512 (PMC3412845; doi:10.1371/journal.pone.0042512)
Supplement: Table S1 — A comparison of BS potencies at native GABAA receptors versus recombinant receptors of different compositions expressed in HEK 293 cells. Recombinant GABAA receptors were activated with the maximal GABA concentration (50–1000 µM) except (*) for the β3 homopentameric receptors, where amplitudes of outward currents, evoked by UDCA application, were analysed. As multiple applications of UDCA in these experiments caused run-down of block of spontaneous channel openings, IC50 is roughly estimated (∼). All IC50s values were compared with those obtained for the juvenile mouse neurons. Only in case of the mutated α1 (V256S) subunit the potency of UDCA was significantly different. (DOC) [file pone.0042512.s004.doc]

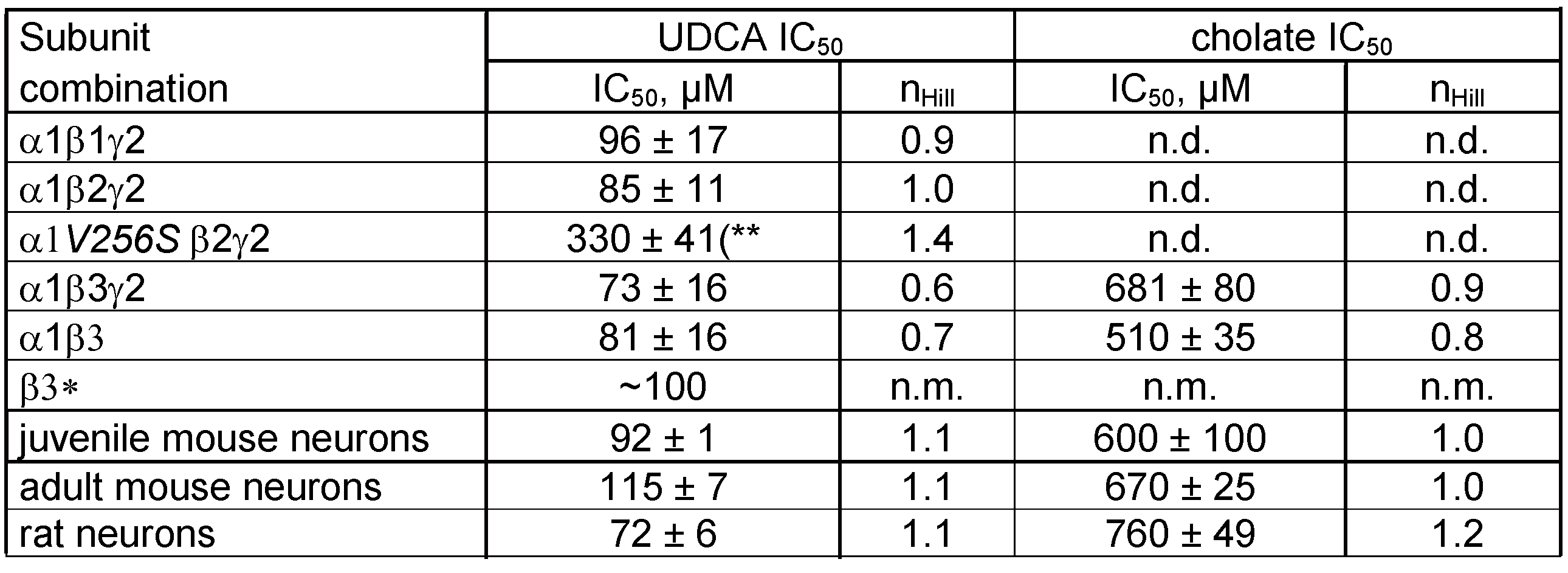


**Supplementary Table 1.** A comparison of BS potencies at native GABA_A_ receptors versus recombinant receptors of different compositions expressed in HEK 293 cells.

Recombinant GABA_A_ receptors were activated with the maximal GABA concentration (50 -1000 µM) except (*) for the β3 homomeric receptors, where amplitudes of outward currents, evoked by UDCA application, were analysed. As multiple applications of UDCA in these experiments caused run-down of block of spontaneous channel openings, IC_50_ is roughly estimated (~). All IC_50_s values were compared with those obtained for the juvenile mouse neurons. Only in case of the mutated α1 (V256S) subunit the potency of UDCA was significantly different
